# Mycosis fungoides with spongiosis: a case report

**DOI:** 10.1186/s13256-023-04188-2

**Published:** 2023-11-03

**Authors:** Jesse Jonathan Kashabano, Eulade Rugengamazi, Subira Bhoke Matiku, Rashid Mussa Mruma, Emmanuel L. Lugina

**Affiliations:** 1 Department of Clinical Oncology, Muhimbili University of Health and Allied Sciences, Dar Es Salaam, Tanzania; 2 Ocean Road Cancer Institute, Dar Es Salaam, Tanzania; 3 Department of Pathology, Muhimbili University of Health and Allied Sciences, Dar Es Salaam, Tanzania; 4 Benjamin Mkapa Hospital, Dodoma, Tanzania

**Keywords:** Case report, Chemotherapy, Total skin electron beam radiotherapy, Mycosis fungoides, Ocean Road Cancer Institute

## Abstract

**Background:**

Mycosis fungoides (MF) is the most common form of cutaneous T-cell lymphoma (CTCL). CTCL are an uncommon, heterogeneous group of non-Hodgkin lymphomas (NHLs) of T- and B-cell origin where the skin is the primary organ of involvement. It is characterized by malignant CD4^+^ T-cells infiltrating the skin and other organs, leading to progressive skin and systemic involvement. Histopathologically, MF is characterized by atypical lymphocytes demonstrating epidermotropism without spongiosis. Spongiosis is the histological hallmark of intercellular epidermal edema, viewed as clear spaces within the epidermis, and is very common in benign inflammatory dermatoses. Very few studies have reported MF in sub-Saharan Africa (SSA). We are reporting a case of MF with a rare presentation of spongiosis treated successfully with a low dose total skin electron beam therapy (TSEBT) followed by maintenance therapy of low dose Methotrexate (MT) at the Ocean Road Cancer Institute (ORCI) in Tanzania. This is the first case of MF to be managed with low-dose TSEBT in Tanzania. The authors wish to create awareness of the disease among physicians and pathologists and expand on the data paucity in SSA.

**Case description:**

We are reporting a case of a 31-year-old male of African origin who self-referred to our oncology center with a 4-year history of skin rashes throughout the body, which was unresponsive to topical steroid treatment. The biopsy was taken, and the patient was diagnosed with MF CD 3 positive with spongiosis. The patient was treated with radiotherapy, whereby he received low dose total skin electron beam therapy (TSEBT) 12 Gy in 3 fractions at a daily dose of 4 Gy, followed by maintenance therapy of low dose Methotrexate and attained an excellent therapeutic response.

**Conclusion:**

Spongiosis is an infrequent presentation of MF. Low-dose TSEBT provides reliable and rapid reduction of disease burden in patients with MF, which could be administered safely multiple times during a patient's disease with an acceptable toxicity profile. Lack of tendency to perform skin biopsies and cost constraints in assessing multiple immunophenotypic markers lead to missing the diagnosis. Diagnosis and treatment of MF in resource-limited countries is challenging.

## Introduction

Cutaneous T-cell lymphomas (CTCLs) encompass a heterogeneous collection of non-Hodgkin lymphomas (NHL) that arise from skin-tropic memory T lymphocytes. Among them, mycosis fungoides (MF) and Sézary syndrome (SS) are the most common malignancies [1]. The disease is named MF due to its histological resemblance to a fungal infection [2].

MF is the most common variant of primary CTCL, accounting for 50% of all primary CTCL, and represents less than 1% of the total number of NHL [3]. MF is characterized by malignant proliferation of CD4 + T cells with epidermotropism in the skin and, generally, has a prolonged indolent clinical course [4]. The disease has a male-to-female ratio of 2:1. It can occur in any age group but is most commonly diagnosed in middle-aged and older individuals, with a median age of 60 years at diagnosis. MF has a higher incidence in specific populations, such as African Americans, who have a 2–3 times greater risk of developing the disease than whites [5]. Very few studies have reported on MF in sub-Saharan Africa (SSA) [6–10].

Several issues, including Human Immunodeficiency Virus (HIV) co-infection, lack of access to specialists, and a relatively large dispersed rural population, may contribute to the burden and late presentation of CTCL seen in SSA. The literature suggests that CTCL is likely under-recognized and under-diagnosed in SSA [6–8].

MF usually presents as patches, plaques, nodules, or tumors according to the stage of the disease [11]. Clinicians must consider the diagnosis of CTCL early when presented with a persistent chronic dermatitis resistant to initial treatments or featuring morphologies such as erythroderma, nodules, tumors, and lymphadenopathy [9].

MF is classified into four clinical stages according to the TNMB classification (tumor-node-metastasis-blood) that takes into account the extent of cutaneous involvement based on the body surface, the presence of nodal or visceral disease, and the presence of Sezary cells at the peripheral blood level (Table 1) [3].

**Table 1:** Showing TNMB staging of MF

T stage	N stage
T1: Limited patches, papules, and plaques covering 80% of body surface area T2: Patches, papules, or plaques covering ≥ 10% of the skin surface. May further stratify into T2a (patch only) vs. T2b (plaque/patch) T3: One or more tumors (≥ 1-cm diameter) T4: Confluence of erythema covering > 80% body surface area	N0: No clinically abnormal peripheral lymph nodes; biopsy not required N1: Clinically abnormal peripheral lymph nodes; histopathology Dutch grade 1 or NCI LN0–2 (N1a, clone negative; N1b, clone positive) N2: Clinically abnormal peripheral lymph nodes; histopathology Dutch grade 2 or NCI LN3 N3: Clinically abnormal peripheral lymph nodes; histopathology Dutch grades 3 or 4 or NCI LN4; clone positive or negative NX: Clinically abnormal peripheral lymph nodes; no histologic confirmation

B stage	M stage
B0: Absence of significant blood involvement: < 5% of peripheral blood lymphocytes are atypical (Sézary) cells B1: Low blood tumor burden: > 5% of peripheral blood lymphocytes are atypical (Sézary) cells but do not meet B2 criteria B2: High blood tumor burden: ≥ 1000/mL Sézary cells with positive clone	M0: No visceral organ involvement M1: Visceral involvement (must have pathology confirmation, and the organ involved should be specified)

Staging groups	Staging groups
IA: T1 N0 M0 B0–1 IIIA: T4 N0–2 M0 B0 IB: T2 N0 M0 B0–1 IIIB: T4 N0–2 M0 B1	IIA: T1–2 N1–2 M0 B0 IVA1: T1–4 N0–2 M0 B0–2 IIB: T3 N0–2 M0 B0–1 IVA2: T1–4 N3 M0 B0–2 IVB: T1–4 N0–3 M1 B0–2

This is the first case of MF to be managed with low-dose TSEBT in Tanzania. The authors wish to create awareness of the disease among physicians and pathologists and expand on the data paucity in SSA.

## Case presentation

A 31-year-old male teacher of African origin presented to Ocean Road Cancer Institute (ORCI) with a four-year history of an itchy rash on the face, trunk, and limbs. The rash initially appeared as macules and papules over the trunk, 2–3 in number. After several months, the lesions gradually progressed to involve limbs, buttocks, face, and the entire trunk. The rash eventually ulcerated. He had received multiple topical treatments (steroids and moisturizing creams) as an eczema case from 2017 to 2020 without a clinical response. He denied a similar illness in the past and had no history of chronic diseases. There was no history of exposure to occupational chemicals, drugs, radiation, or infections before the appearance of the lesions, and none of the family members had a similar illness or history of atopy. The patient neither took any form of alcohol nor tobacco products. The rash was associated with on-and-off diarrhea episodes, though denied a history of evening fevers, night sweats, loss of weight, or arthralgia.

On examination, the patient was alert, afebrile, not dyspneic, not pale, not jaundiced, and had no lower limb edema, vitals, blood pressure 137/81 mmHg, pulse rate 87 beats per minute, respiratory rate 15 breaths per minute, oxygen saturation 98%. He had a functional status of ECOG 1. On local examination, multiple well to ill-defined hyperpigmented erosive plaques, nodules, and tumors were present on the trunk, all limbs, and face, occupying more than 95% of the body surface area, including the non-sun-exposed location. The lesions had various sizes ranging from 6 cm in the widest dimension to 2 cm. There was an ulcer of about 8*10 cm on the left knee and a small ulcer on the left elbow, approximately 2*3 cm (Figs. 3 and 4). There was no significant peripheral lymphadenopathy or splenomegaly. On systemic and neurological examinations, the patient had a GCS of 15/15, oriented to time, people, and place, with normal speech and good long- and short-term memory. Cranial nerves (1-XII) were all intact, and no signs and symptoms of meningeal irritation, with normal tone and reflexes with power of 5/5 on both lower and upper limbs; superficial and deep tendon reflexes were normal, sensory system intact with normal coordination, gait and balance. Cardiovascular System: S1 and S2 were heard with no added sound. Respiratory system: normal chest cavity with good air entry, no crackles or wheezes heard. Abdominal examination: normal abdominal contour, no palpable mass, with normal bowel sounds. The differential diagnoses were atopic dermatitis (AD), chronic eczema, and psoriasis.

**Fig. 1:**
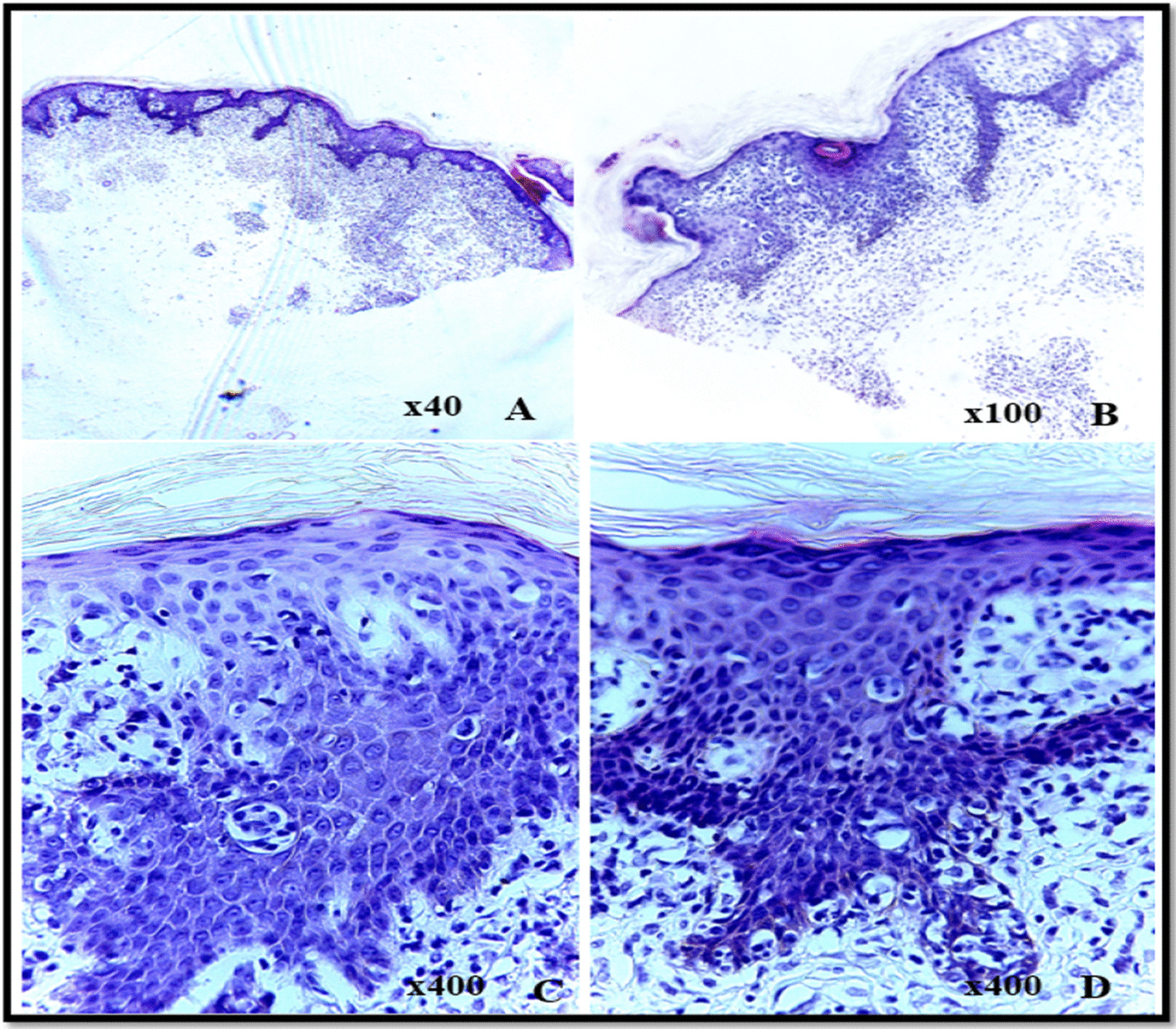
Photomicrographs of Hematoxylin and Eosin-stained sections showing a skin biopsy: **A** and **B** hyperkeratotic skin with extensive band lymphocytic inflammatory infiltrate in the papillary and reticular dermis. **C** and **D** Acanthosis and prominent spongiosis, Note the epidermotropism of the hyperchromatic lymphocytes with cerebriform nuclei, as well as occasional Pautrier microabscesses. Blood investigations: Complete blood count: WBC 8.67 109/L; 8.6 g/dL; HCT 26.4%; MCV 58.6 fL; NEU 6.39 10 3/μL; PLT 656.0 103/μL, Liver function tests: ALT 47.50 IU/L; AST 21.8000 IU/L and Renal function test: Serum creatine 84.170 µmol/L

A skin biopsy revealed MF with prominent spongiosis (Fig. 1). Immunohistochemistry showed CD3 positivity. Immunohistochemistry for CD4, CD5, and CD8 was not done.

Urinalysis and Microbiology were not done.

**Fig. 2:**
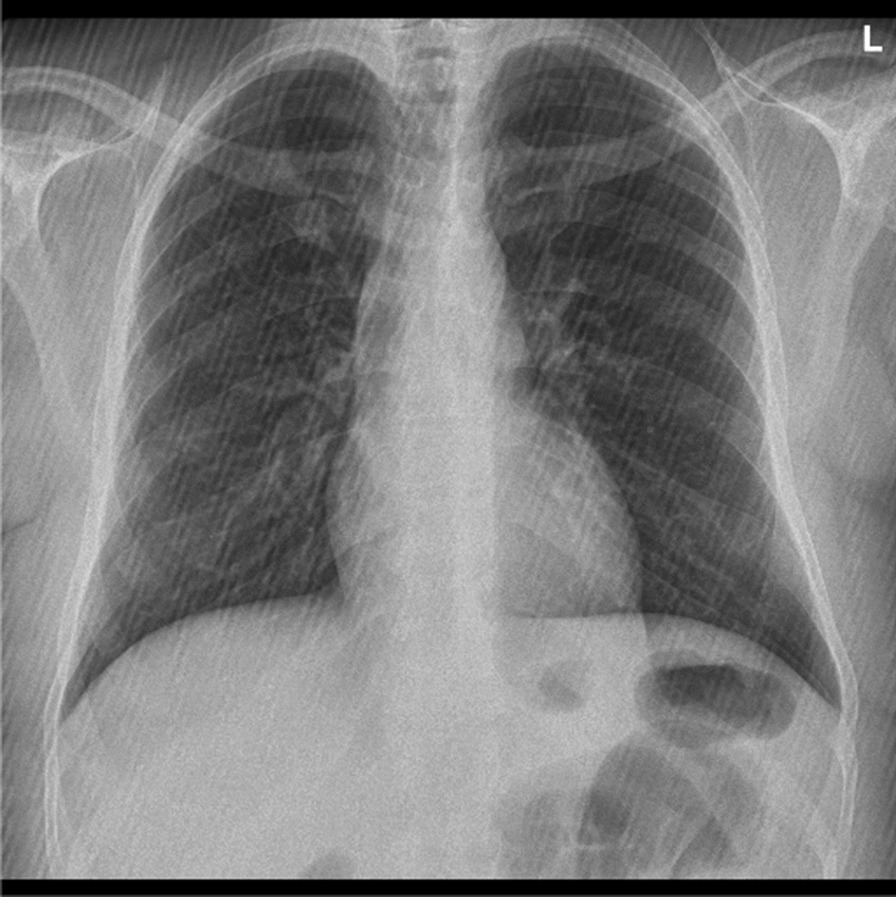
Showing the normal chest x-ray

HIV1 serology test was negative. Peripheral blood smear and bone marrow aspirate were not done. Chest x-ray and abdomen ultrasound were normal (Fig. 2).

Upon completion of this evaluation, the patient was scored to have 6 points (Persistent and progressive patches/thin plaques-2 points; Non-sun-exposed location and Size/shape variation-2 points; Superficial lymphoid infiltrate-1 point; Lymphocytic atypia-1 points and CD3 + − 1 point) according to International Society for Cutaneous Lymphomas (ISCL) diagnostic algorithm [4] and the diagnosis of MF was rendered. The TNMB stage was stage 3 (T3N0M0BX) according to the International Society for Cutaneous Lymphoma (ISCL) and the Cutaneous Lymphoma Task Force of the European Organization of Research and Treatment of Cancer (EORTC) [3].

The initial treatment after being admitted was oral Prednisolone 10 mg b.i.d for 30 days and oral Ampiclox capsules 500 mg q.i.d × 10 days.

**Fig. 3:**
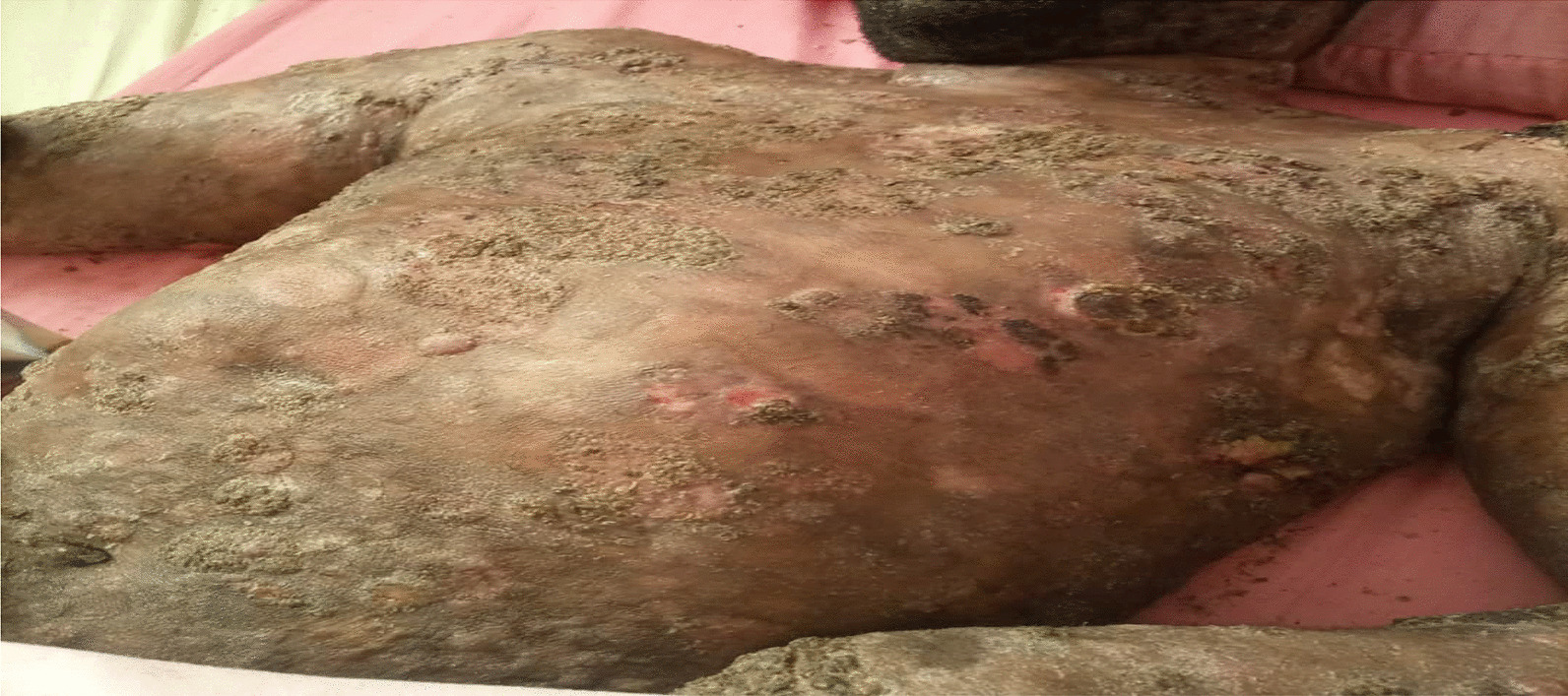
Showing the initial presentation of the patient’s upper trunk

**Fig. 4:**
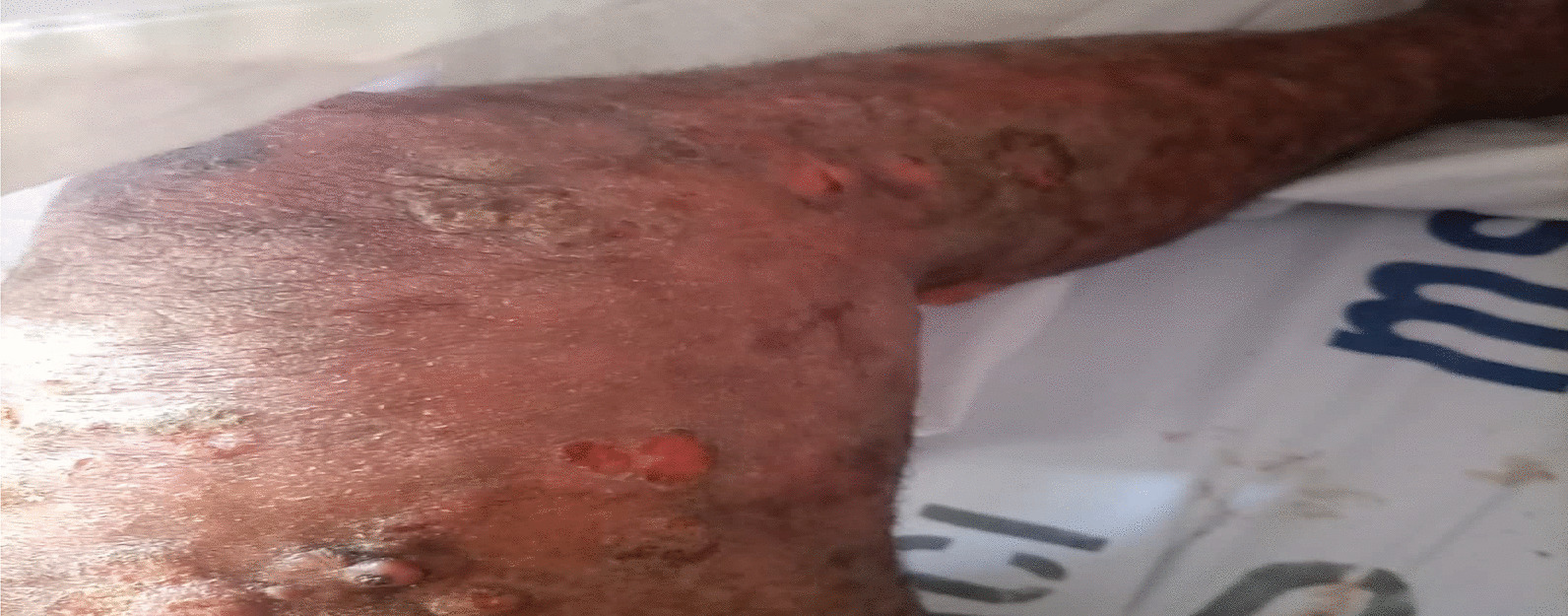
Showing the initial presentation of the patient’s lower trunk

The patient was after that treated with a low-dose TSEBT. He received 12 Gy in 3 fractions at a daily dose of 4 Gy in October 2021. The radiotherapy technique was 3D conformal radiotherapy (3DCRT) using a linear accelerator (LINAC). The patient stood upright and half-naked during treatment, exposing all the skin to radiation. A 12 MeV beam was used to treat this patient using three anterior and posterior fields. The fields were then separated into Upper and Lower half bodies to ensure coverage of the open field size. The gantry angles were 108^0^ and 72^0^ for the upper and lower halves, respectively. The source-to-skin distance was 110 cm, and the field size was 25 × 25 cm (Figs. 3, 4).

**Fig. 5:**
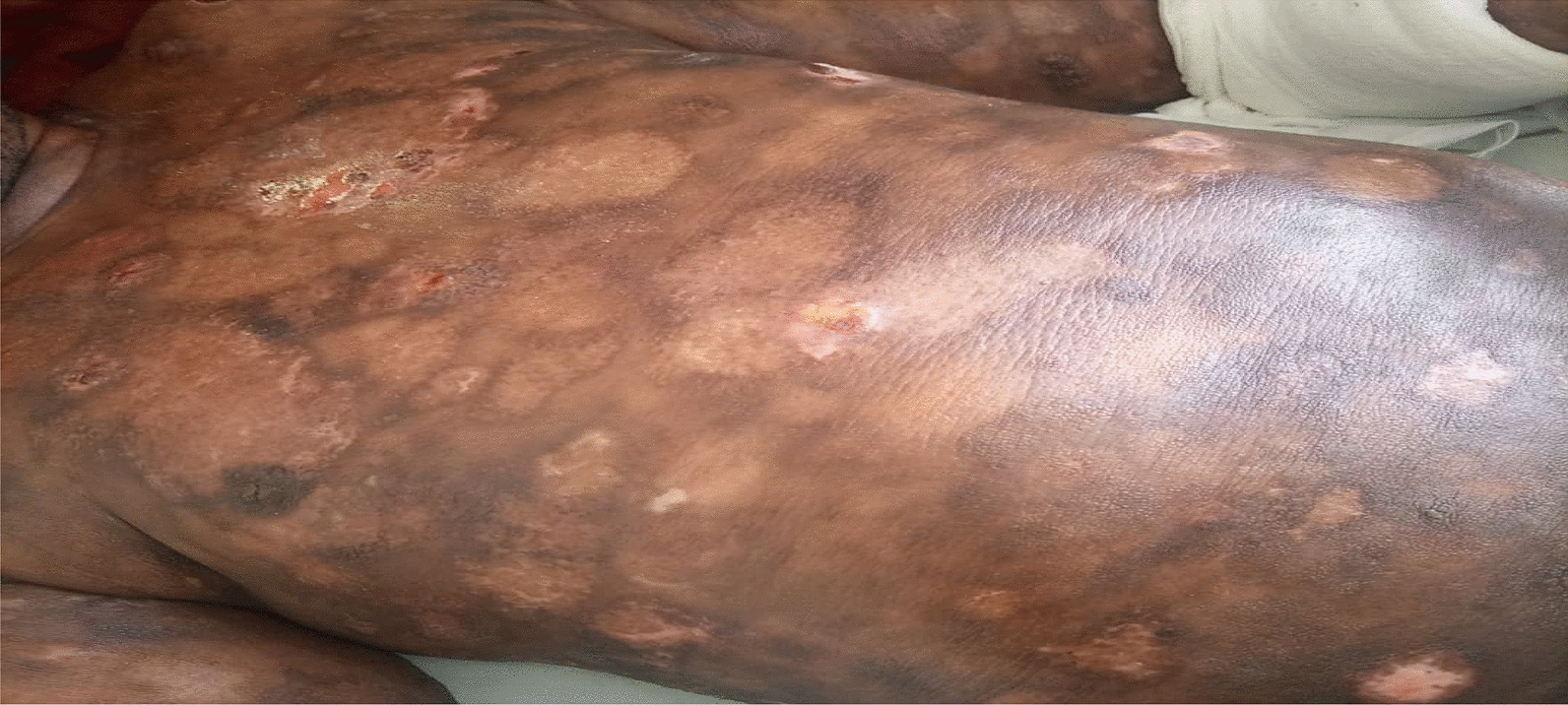
Showing the progress of the patient’s upper trunk (2 weeks post TSEBT)

**Fig. 6:**
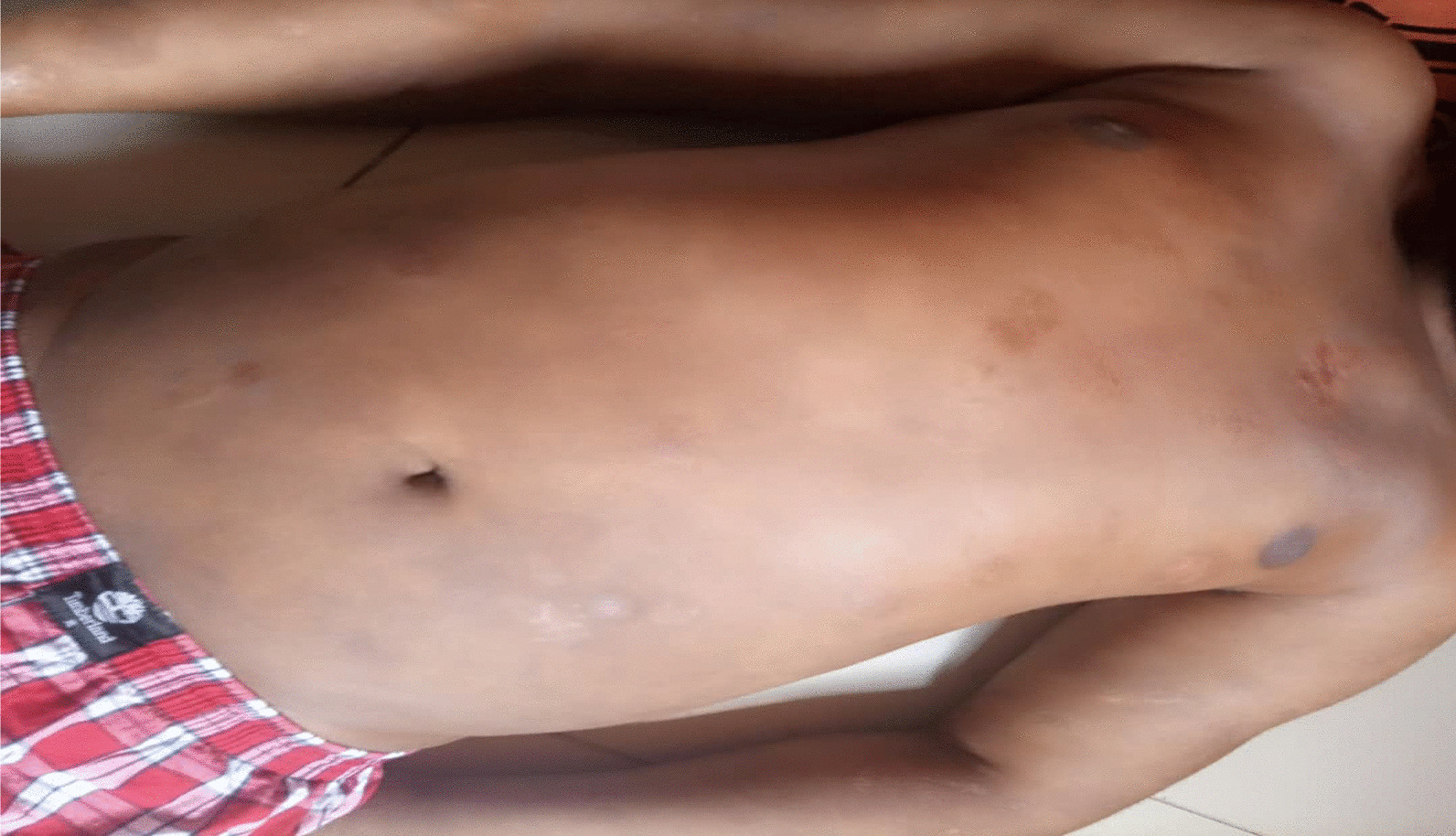
Showing the progress of the patient’s upper trunk (1-month post-TSEBT)

After three sessions of TSEBT, the patient had remarkable improvement in lesions, itching, and pain subsided (Fig. 5). After completion of TSEBT, he was put on maintenance oral methotrexate (50 mg weekly with 1 week of rest after every 4 weeks). He attained a complete response (CR) of all the lesions within three months after completing TSEBT (Fig. 6). He did not experience any acute or late effects of radiotherapy.

**Fig. 7:**
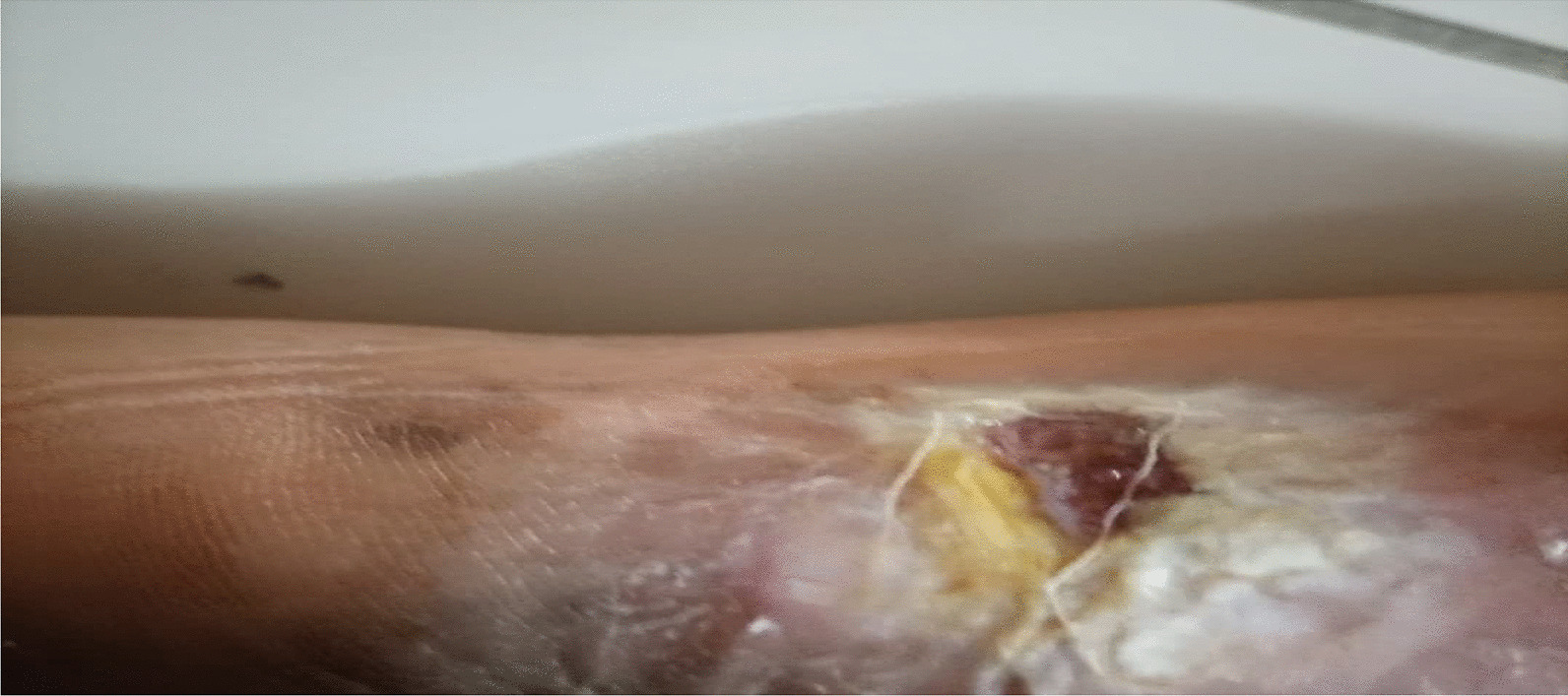
Showing recurrent lesion (6 months post TSEBT)

**Fig. 8:**
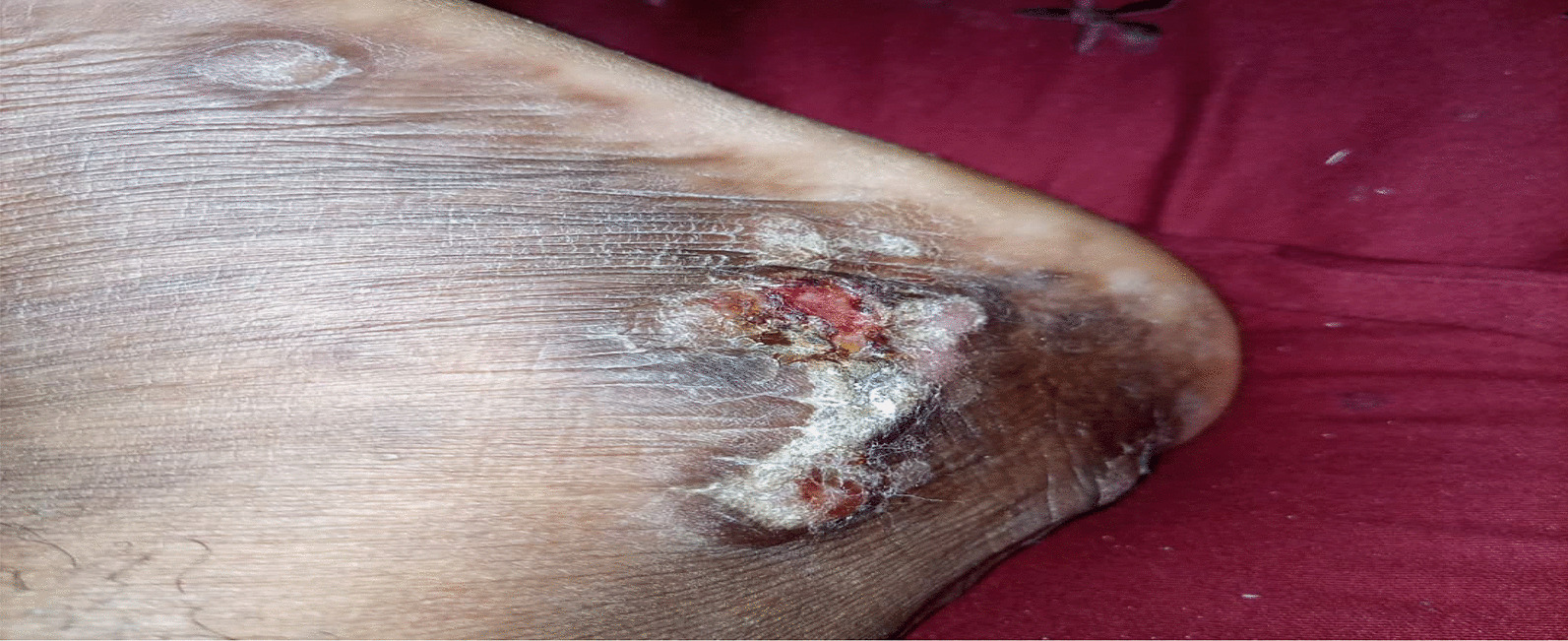
After radiotherapy

Five months later, the patient developed a recurrence lesion in his left leg (Fig. 7). He was treated with localized TSEBT (12 Gy in four fractions) with a complete response (Fig. 8).

**Fig. 9:**
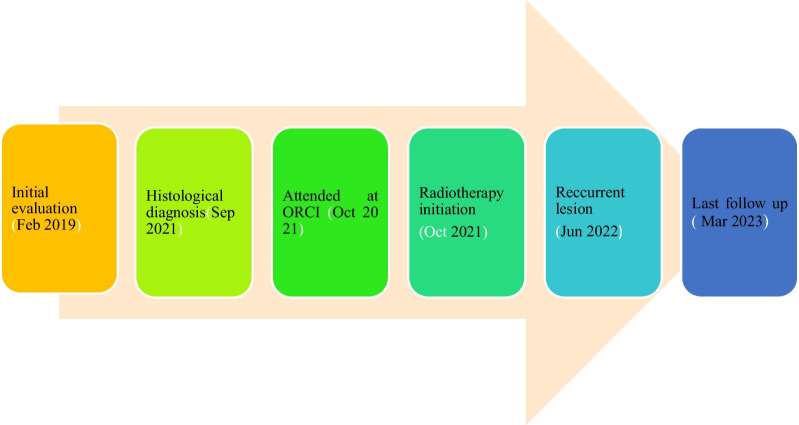
A timeline of significant events

The patient is in remission two years post initial treatment and is still on maintenance oral methotrexate 50 mg weekly. Summary of important events have been highlighted (Fig. 9).

## Discussion

We are reporting a case of advanced MF with spongiosis with a complete, durable response after low-dose total skin electron beam therapy (TSEBT) and challenges in managing MF in resource-limited countries. Spongiosis and durable response after treatment are very rare in MF.

The cause of MF remains largely unknown. Previous studies suggest that superantigens might be involved in the pathogenesis of MF. Persistent infections with pathogenic microbes that are poorly managed may provide an antigenic trigger for MF tumorigenesis. Therefore, the high prevalence of multiple co-existing viral and/or bacterial microorganisms in African populations, like HIV, HTLV-1/2, Epstein-Barr virus (EBV), Cytomegalovirus (CMV), Human Herpesvirus-8, and Staphylococcus aureus might play a significant role, since they are known to provide chronic immune stimulation inducing T-cell proliferation and possibly neoplastic transformation [12]. However, the association between MF and HTLV-1/2 is controversial [13].

The diagnosis of MF is often challenging, especially in resource-limited countries, mainly because of the atypical clinical presentation at an early stage [14]. Indeed, as in the case of our patient, lesions can simulate psoriasis, atopic dermatitis, or chronic eczema. The diagnosis of MF is challenging even in resource-abundant countries. An extended follow-up, as seen in our patient, and evaluation of multiple biopsies are often necessary to confirm the diagnosis. Before biopsy of an involved early-stage skin lesion, therapy is typically withheld for 2–4 weeks since the treatment effect can obscure the pathognomonic histologic features, including epidermotropism and the ‘cerebriform’ nuclear appearance. To facilitate the diagnosis of MF in HIC, an algorithm was proposed by the International Society for Cutaneous Lymphomas (ISCL) diagnostic algorithm [4]. The algorithm holistically integrates clinical, histopathologic, immunopathologic, and molecular biological characteristics. One of the histopathological criteria is epidermotropism without spongiosis [4]. Spongiosis represents the histological hallmark of intercellular epidermal edema, translated by cell condensation with corresponding wide spaces between keratinocytes, with elongated intercellular bridges (‘spinous processes’), leading to a sponge‑like appearance of the epidermis [15]. Hematoxylin and eosin (H&E) routine staining highlights this process as clear spaces within the epidermis, and the most common cutaneous lesion associated with spongiosis is eczematous dermatitis [15].

Our patient was diagnosed with MF with spongiosis, mimicking benign inflammatory dermatoses. Lack of awareness of spongiosis as a rare presenting feature of MF may result in initial misdiagnosis as a benign inflammatory condition [16].

Since unaffordability and lack of access preclude the assessment of multiple immunopathological and molecular markers in SSA, a consensus was reached that in resource-limited settings, the combination of characteristic clinical findings, morphologic appearance on hematoxylin and eosin staining, and a positive CD3 or CD4 immunohistochemical result is sufficient for diagnosing MF [7].

In addition to overcoming these challenges associated with diagnosing MF, an additional potential neoplasm, adult T-cell leukemia/lymphoma (ATLL), should be considered when diagnosing MF in Africa. Human T-cell lymphotropic virus 1 (HTLV-1) is endemic in parts of Africa, Japan, and the Caribbean and was first identified in cells from a patient diagnosed with CTCL [17]. Since this discovery, a smoldering clinical subtype of ATLL of the skin was described that can mimic CTCL clinically and pathologically [18]. A diagnosis of smoldering ATLL requires either the presence of more than 5% abnormal circulating T-lymphocytes in the peripheral blood or histological confirmation of typical ‘flower cells’ in a T-cell infiltrate not seen in our patient [19]. A serologic test for HTLV-1 can help differentiate the smoldering ATLL of the skin from CTCL. Immunohistochemistry for CD25 may also be helpful, as it is expressed strongly in almost all cases of ATLL [7]. East Africa, including Tanzania, appears globally much less endemic for HTLV-1 than West and Central Africa [20], and therefore, no additional tests were done to rule out HTLV-1 in our patient.

MF is generally considered an incurable disease, but it should be noted that most patients have indolent forms classified as stage IA or IB in 65 to 85% and are, therefore, long-term survivors [21]. Patients with early stages (IA, IB, IIA) often initially receive skin-directed therapies, including topical corticosteroids, topical retinoids, phototherapy, topical chemotherapy, and radiotherapy. At the same time, advanced-stage (IIB, III, IV) is treated with systemic therapy [7]. There is insufficient data to recommend one treatment regimen over another. While several treatment guidelines for CTCL exist in resource-abundant regions, this is different for resource-limited settings [7].

Topical skin treatments mainly include systemic or topical corticosteroids, such as betamethasone dipropionate 0.05%, which results in a complete and partial response rate of 60–65% and 30% in the case of T1 and 25 and 57% in the T2 stage, respectively [22]. Topical corticosteroids may be effective in the patchy stage (early stage) MF and not the plaque stage (advanced stage), perhaps due to poor penetration into the reticular dermis, as was observed in the index case. Despite demonstrating the efficacy of corticosteroids in early-stage MF, the above study did not define how the response rates were measured and did not include higher MF stages. A recent study also found a high response rate (81%) to topical steroid monotherapy, mainly clobetasol, in early-stage MF. However, not surprisingly, there was a poor response (33.3%) to topical steroid monotherapy in patients with higher-stage MF (IIA and above) [23]. Tazarotene, a topical retinoid, is another skin-directed therapy that has also been shown to result in a high complete CR (60%) when it is used as monotherapy for at least six months in early-stage MF [24].

Generalized skin-directed therapy is mainly represented by phototherapy (narrowband-UVB) and or PUVA therapy (Psoralen plus ultraviolet A light therapy), which is indicated in patients with more extensive lesions (Thick plaques) [25].

MF is very radio-sensitivity [14], and Radiotherapy (RT) has been used to treat localized/limited lesions of MF since 1902 [26]. Total skin electron beam therapy (TSEBT) is the most effective skin-directed MF therapy. TSEBT for patients with CTCL is technically challenging, and numerous approaches have been developed to overcome the many fields matching problems associated with such a large and complex treatment volume [27]. Historically, TSEBT doses were escalated from 8 to 36 Gy as tolerance for higher doses was demonstrated, and complete response (CR) rates increased [28]. Toxicity is dose-dependent, with common acute effects being erythema and dry desquamation. Late effects of 36 Gy include temporary anhidrosis and temporary loss of fingernails and toenails. Alopecia may be permanent at doses of more than 25 Gy [29]. Recurrence is very common, even after 36 Gy. There has been a reluctance to re-treat MF with more than two courses of 36 Gy TSEBT because of the risk of significant skin atrophy and xerosis [30]. The recurrence-free interval after RT ranges from 30 to 60 months [10]. There is thus a renaissance of low-dose TSEBT. Advantages of low-dose TSEBT include a shorter treatment duration, reduction of toxicities, reduced cost, particularly in resource-limited countries, improved patient compliance, and the ability to use the therapy more frequently over the patient’s lifetime. Lower doses may also allow the use of systemic radiosensitizers [31].

In a study by Wilson *et al.*, a complete response (CR) rate of 97% was observed. Notably, when examining recurrence rates in relation to different total doses of local superficial radiation, they found that a local recurrence rate of 25% was associated with treatment to a dose of 20 Gy. However, this local recurrence rate decreased to 8% when treatment doses ranged from 20 to 40 Gy [32]. Similarly, the study by Cotter *et al.*, showed that the local recurrence rate was 42% for lesions treated with doses of 10 Gy or less. However, when the lesions were treated with doses exceeding 30 Gy, the local recurrence rate dropped to 0% [33].

The main therapeutic options for early-stage CTCL in resource-limited settings include topical corticosteroids, radiation therapy, and heliotherapy; the latter may be used instead of phototherapy, which is not usually available [7].

Heliotherapy (HT) or sunbathing is a light-based therapy proven to be convenient for alleviating inflammatory skin changes. The UV lights reach the epidermis and underlying dermis, triggering immunosuppression, enhancing apoptosis, suppressing cellular proliferation, and inducing vitamin D production. Furthermore, it is considered that HT has strong antipruritic effects [34]. The optimal dose of HT has never been specifically studied in CTCL [7]. The dose can be estimated from a South African study that measured the natural UVB exposure at meteorological centers in Cape Town, Durban, and Pretoria throughout the year. In patients with Fitzpatrick skin type VI, 0.08 J/cm^2^ is the initial recommended daily treatment dose (0.03 J/cm^2^ in type III skin) for CTCL. Previous studies showed that 70% of the minimal erythema dose (MED) is effective in treating MF, with the daily dose increased by 15% every three days until a maximally tolerated dose (MTD) is reached [35]. This MTD is then maintained throughout the treatment course. To get MTD with HT, a patient in Pretoria at 10 am with type VI skin, for example, requires 77 minute of HT to all affected areas in December, 113 minute in March, and 228 minute in June (one-third of this duration is needed for type III skin in each instance) [36]. These durations of HT may be overestimated for an equatorial country like Tanzania, with less seasonal variation and more intense UVB exposure. However, much time is still required to achieve therapeutic UVB doses of HT, especially in dark-skinned individuals [6]. HT has been reported to result in partial response among patients with MF in Botswana [9].

Refractory early-stage or advanced-stage MF requires systemic therapy and a multidisciplinary approach. Various combinations of skin‐directed therapies, systemic retinoids, histone deacetylase inhibitors, interferon-alpha, antibody–drug conjugates (Brentuximab vedotin), monoclonal antibodies (Mogamulizumab), and ultimately chemotherapeutic agents and hematopoietic stem cell transplants are used in the management of these patients [37]. The management of MF in resource-limited countries is complex because of the unavailability of most therapies except steroids, chemotherapeutic agents, radiotherapy, and heliotherapy.

The low-dose TSEBT has the highest overall response rate (ORR) (88%) and complete response rate (CR) (32%) of all skin-directed therapies. ORRs associated with topical corticosteroids, nitrogen mustard, and narrowband ultraviolet B are reported to be 57%, 72%, and 75%, respectively. Response rates to systemic agents such as Bexarotene and Vorinostat (histone deacetylase inhibitor) are 51% and 31%, respectively, with less than 10% CR rates [31]. Weekly methotrexate (MTX) with doses ranging from 25 to 50 mg results in CR of 12% in T2 patients and a 22% PR rate with a median recurrence-free interval of 15 months. Weekly MTX is of limited value in advanced-stage MF [38]. Liposomal doxorubicin has an ORR of 56% (20% CR) [39]. Gemcitabine 1000 mg/m^2^ days 1, 8, and 15 q4 weeks has an ORR of 68% (8% CR) [40]. Despite the highest CR of TSEBT, the clinician’s awareness of this treatment remains low because of concern about side effects [27].

Our patient was put on weekly oral MTX as maintenance therapy; however, until today, there are no standardized strategies for maintenance therapy in patients with CTCL who achieved disease control [41].

## Conclusion

Spongiosis is an infrequent presentation of MF. Low-dose TSEBT provides reliable and rapid reduction of disease burden in patients with MF, which could be administered safely multiple times during a patient’s disease with an acceptable toxicity profile. Diagnosis and treatment of MF in resource-limited countries is challenging.

## Data Availability

Not applicable.

## Abbreviations

NHL: Non-Hodgkin’s lymphoma
CTCL: Cutaneous T-cell lymphoma
LINAC: Linear accelerator
MF: Mycosis fungoides
ORCI: Ocean Road Cancer Institute
OS: Overall survival
TSEBT: Total skin electron beam therapy
OSS: Overall response rate
